# Predictors of Phosphodiesterase Type 5 Inhibitor Treatment Failure in Patients Diagnosed With Erectile Dysfunction

**DOI:** 10.7759/cureus.50515

**Published:** 2023-12-14

**Authors:** Muath Albarakati, Hossam S El-Tholoth, Abdulaziz Alzahrani, Omar S Alghamdi, Abdulrahman Alquliti, Musa Alnuami, Abdalmagid Althobity, Abdulmalik Almardawi, Khaled Bedaiwi

**Affiliations:** 1 Urology, Armed Forces Hospital-Al Hada, Makkah, SAU; 2 Urology, Prince Sultan Military Medical City, Riyadh, SAU; 3 College of Medicine, King Saud University, Riyadh, SAU; 4 Urology, King Fahad Hospital, Albaha, SAU

**Keywords:** high hba1c, low free testosterone, follow-up, non-responder, phosphodiesterase type 5 inhibitors, erectile dysfunction

## Abstract

Introduction: Erectile dysfunction (ED) is a prevalent condition, especially in aging populations, with significant implications for quality of life. While phosphodiesterase type 5 inhibitors (PDE5Is) are the first-line treatment, a substantial percentage of patients do not respond satisfactorily. This study aimed to identify predictors of PDE5I treatment failure in ED patients.

Methods: Data from January 2016 to January 2022 was reviewed for patients with ED who either failed PDE5I treatment or had a successful outcome. Demographic, medical, and laboratory data were collected and analyzed. Patients with contraindications or who did not complete the treatment were excluded.

Results: The treatment failure group comprised 288 patients, while 225 age-matched patients formed the control responder group. There were no significant differences in marriage duration, comorbidities, smoking, or BMI. However, HbA1c levels, free testosterone, and SHBG were significantly different between the two groups.

Conclusion: This study found that high HbA1c levels, low free testosterone, and high sex hormone-binding globulin (SHBG) were associated with PDE5I treatment failure. Managing high HbA1c levels and addressing hormonal imbalances may enhance PDE5I treatment effectiveness in ED patients. However, further research is needed to explore other factors contributing to treatment outcomes.

## Introduction

Erectile dysfunction (ED) can occur at any age but is more likely after the age of 40, as it affects 10−15% of men aged 40−49 years, 20−40% of men between 60 and 69 years old, and 50−100% of men aged 70 and above [1]. ED is defined as a persistent and recurrent inability to achieve and maintain a sufficiently firm penile erection for satisfactory sexual intercourse [2]. As this is an age-related issue, given the rapid population aging, it is expected to affect 322 million individuals worldwide by 2025 [3].

However, extant research states that pohosphodiesterase type 5 inhibitor (PDE5I) treatment contributes to a remarkable recovery of erection. Still, in 20-30% of men, orally-administered PDE5I fails to produce satisfactory results [4]. In 30-50% of such cases, according to some authors, intracavernosal injections of erectogenic drugs, intraurethral alprostadil, or the invasive insertion of a penile prosthesis can yield beneficial results, even though these treatment modalities are liable to more adverse side effects than PDE5I [5].

Guided by this evidence, the present study aimed to determine the most reliable predictors of PDE5I failure in patients affected by ED to improve treatment outcomes and patient compliance.

This article was previously posted to the Research Square preprint on September 28, 2023.

## Materials and methods

After Institutional Review Board (IRB) approval (Approval number: E-2153), data for this retrospective study was gathered by reviewing our hospital records for the period spanning from January 2016 to January 2022, focusing on patients with ED in whom PDE5I treatment failed, due to which they subsequently received intracorporeal injections or were booked for penile prostheses. We also identified age-matched patients for whom PDE5I was beneficial to facilitate comparisons and allow the identification of factors that influence treatment outcomes. According to the management protocol in place at our hospital, patients experiencing ED should be given PDE5Is (Sildenafil 50 mg when needed, Tadalafil 20mg when needed) for at least eight weeks. In addition, they should be reevaluated at six-monthly intervals to assess their response and compliance with the treatment, based on which the PDE5I dose and type should be adjusted as required. The protocol was unified across all cases. Patients in whom treatment failed and those that required the adoption of the second and/or third line of treatment were of interest for the present investigation and defined as the PDE5I treatment failure group, and thus formed the study sample. Unmarried patients, patients with Peyronie’s disease, patients who have not completed the course of treatment or did not comply with dose modification, patients with contraindications for PDE5Is or at risk of adverse events (such as patients with a recent history of stroke or myocardial infarction, individuals with low or high blood pressure, as well as those with unstable angina, severe cardiac failure, severe liver impairment, or end-stage kidney dysfunction requiring dialysis), all patients received other oral supplement and/or hormonal treatment, all patients submitted to radical prostatectomy or other radical pelvic surgeries or radiation therapy, and those who refused PDE5I treatment or developed side-effects related to PDE5Is were excluded.

When reviewing the medical records of patients that met the study inclusion criteria, demographic information (age, marriage duration, comorbidities, smoking status, and body mass index [BMI] obtained during the first clinic visit) was gathered and grade 0 was assigned to those without any comorbidities, whereas grades 1−3 indicated the presence of one, two and at least three chronic medical conditions, respectively. At this stage, we recorded the findings yielded by initial laboratory evaluation tests, including HbA1c, total testosterone (TT), free testosterone (FT), sex hormone-binding globulin (SHBG), estradiol, ollicle-stimulating hormone (FSH), luteinizing hormone (LH), prolactin, vitamin D, cholesterol, low-density lipoprotein (LDL), high-density lipoprotein (HDL), triglyceride, prostate-specific antigen (PSA), thyroid-stimulating hormone (TSH), thyroxine (T4), and hemoglobin (HB) level. This group of patients was considered the treatment failure group and was compared to the control group, comprising age-matched patients in whom PDE5I treatment yielded satisfactory results.

As per our protocol, venous blood samples were collected from each patient between 8 AM and 11 AM, following an overnight period of fasting. TT levels were measured using the electrochemiluminescence immunoassay (ECLIA), which is intended for use on Cobas E immunoassay analyzers. Although SHBG levels were determined, the ECLIA is intended for use on Cobas E immunoassay analyzers. FT levels were measured using the ETI-MAX 3000 instrument.

All analyses were conducted using SPSS version 20 (IBM Corp., Armonk, NY) commercial software. When analyzing patient data, the mean and standard deviation were calculated for continuous variables while reporting relative frequencies for categorical variables. Student’s t-test and analysis of variance (ANOVA) were performed to compare means, while the chi-squared test was used for comparisons involving categorical data. The variables that emerged as statistically significant (at p < 0.05) were used to design a logistic regression model. By comparing patients in whom PDE5I treatment was unsuccessful (the treatment group) to those in whom it was beneficial (controls), the aim was to identify the main risk factors that would contribute to treatment failure. Accordingly, the findings could be used to revise the treatment plan or to offer counselling to patients with ED.

## Results

In this study, the treatment group consisted of 288 patients who experienced treatment failure with PDE5Is, while the control group, matched for age, comprised 225 patients. The age distribution in the treatment failure group had a mean age of 65.3 years with a standard deviation of 12.4, whereas the control group had a mean age of 64.6 years with a standard deviation of 12.1.

Table 1 presents the results, revealing no statistically significant differences between the two groups in terms of marriage duration, comorbidities, smoking status, diabetes mellitus (DM), or BMI.

**Table 1:** Comparing the demographic data of the patients with erectile dysfunction that benefitted (control responder group) and did not benefit (treatment failure group) from PDE5I treatment. BMI: body mass index; DM: diabetes mellitus; P-value significant <0.05

Parameters	Treatment failure group (288)	Control responder group (225)	P-value
Age (years) mean ± SD	65.3 ± 12.4	64.6 ± 12.1	0.52
Marriage duration (years) mean ± SD	36.5 ± 8.2	35.7 ± 8.6	0.283
Comorbidities grade 0 N (%)	94 (32.6%)	83 (36.9%)	0.417
Comorbidities grade 1 N (%)	104 (36.1%)	81 (36%)	0.981
Comorbidities grade 2 N (%)	69 (24%)	49 (21.8%)	0.609
Comorbidities grade 3 N (%)	21 (7.3%)	12 (5.3%)	0.359
DM	182(63.2%)	140(62.2%)	0.816
Smoking N (%)	124 (43%)	113 (50.2%)	0.236
BMI (kg/m^2^) mean ± SD	29.7 ± 5.8	29.3 ± 4.5	0.39

However, statistically significant differences were observed between the treatment failure group and the control group in the levels of HbA1c (p < 0.0001), free testosterone (p < 0.0001), and SHBG (p < 0.002), as indicated in Table 2.

**Table 2:** Comparing the laboratory results of the patients with erectile dysfunction that benefitted (control responder group) and did not benefit (treatment failure group) from PDE5I treatment. HbA1c: hemoglobin A1c, SHBG: sex hormone-binding globulin, Estradiol, FSH: follicle-stimulating hormone, LH: luteinizing hormone, LDL: low-density lipoprotein, HDL: high-density lipoprotein, PSA: prostate-specific antigen, TSH: thyroid-stimulating hormone, T4: thyroxine, Hb: hemoglobin. P-value significant <0.05.

Parameters	Treatment failure group (288) mean ± SD	Control responder group (225) mean ± SD	P-value
HbA1c (%)	8.5 ± 1.9	6.8 ± 1.2	<0.0001
Total testosterone (nmol/L)	17.3 ± 7.5	18.4 ± 8.7	0.125
Free testosterone (pmol/L)	30.9 ± 13.3	39.3 ± 23.4	<0.0001
SHBG (mmol/L)	52.6 ± 26.7	45.8 ± 22.9	0.002
Estradiol (pmol/L)	127.9 ± 52.2	120.1 ± 51.1	0.09
FSH (IU/L)	6.9 ± 6.1	5.9 ± 6.3	0.07
LH (IU/L)	8.4 ± 10.6	7.6 ± 8.8	0.36
Prolactin (mIU/L)	198.1 ± 118.3	187.7 ± 119.7	0.33
Vitamin D (nmol/L)	50.7± 28.1	53.3± 30.9	0.32
Cholesterol (mmol/L)	4.2 ± 2.7	4.3 ± 2.3	0.65
LDL (mmol/L)	2.7 ± 0.8	2.6 ± 0.9	0.18
HDL (mmol/L)	1.02 ± 0.30	1.07 ± 0.32	0.07
Triglyceride (mmol/L)	1.6 ± 0.84	1.7 ± 0.91	0.197
PSA (micg/L)	1.7 ± 1.9	1.6 ± 1.8	0.55
TSH (IU/L)	3.1 ± 4.7	3.5 ± 5.1	0.36
T4 (pmol/L)	15.2 ± 2.6	15.3 ±3.6	0.72
HB (g/L)	14.3 ± 1.8	14.5 ± 1.5	0.19

Notably, no statistically significant differences were found between the two groups in the following parameters: TT, estradiol, prolactin, FSH, LH, triglyceride levels, cholesterol levels, LDL levels, HDL levels, PSA levels, TSH levels, T4 levels, vitamin D levels, and Hb levels (Table 2).

## Discussion

Erectile dysfunction is the inability to achieve or sustain penile erection. This condition is influenced by various factors, including neural, psychological, vascular, and hormonal elements [6]. ED diagnosis involves a comprehensive clinical assessment, which consists of a detailed medical/physical examination and a review of the patient’s sexual and psychosocial history [7].

When this cycle is disrupted, men tend to seek medical help and are diagnosed with either ED as a primary condition or as a result of another sexual disorder. Consequently, it is vital to take a detailed sexual history and gather other relevant data (including age, sexual orientation, marital status, and past sexual experiences) to identify the cause of ED and to detect the possible comorbidities, toxic habits, or drugs that could result in ED [8].

In this context, certain risk factors that predispose patients to ED should also be considered, such as dyslipidemia, high blood level, smoking, diabetes mellitus (DM), cardiovascular diseases, excess weight, and a sedentary lifestyle [9]. While ED is highly prevalent, especially in men aged 60 and above, certain risk factors and their role in treatment success are not sufficiently investigated. Given that PDE5Is are considered the first line of treatment for ED, the objective of the present study was to identify additional risk factors that may undermine PDE5I effectiveness. Therefore, following the guidelines for ED diagnosis and treatment, when gathering the data for this retrospective study, patient records were reviewed to obtain all information related to the physical examination of the genitourinary anatomy and the endocrine, as well as the vascular and neurological systems.

We obtained the results of all laboratory tests performed at the patients’ first visit to our hospital, including HbA1c, lipid panel, testosterone level, thyroid function tests, prolactin level, and luteinizing hormone (LH) levels, as advocated by previous studies [10,11]. In addition, given the high prevalence of vitamin D deficiency in our society (estimated at 60-100%) [12], it was also recorded along with the PSA and CBC values.

We focused on patients who had received PDE5I inhibitors and assigned those in whom this treatment was ineffective to the treatment group, while those who benefitted from this intervention served as controls. The decision to focus on this specific treatment mode was guided by an ample body of evidence indicating that PDE5Is are effective in treating ED in a majority of affected men [13].

Our study showed a high level of HbA1c associated with a higher risk of PDE5I treatment failure, as the mean HbA1c level in the control responder group was statistically significantly lower than in the treatment failure group (6.8 ± 1.2 vs. 8.5 ± 1.9, p < 0.0001). In concurrence with what Cayetano-Alcaraz et al. stated, DM seems not only to be the risk factor for developing ED but has also been shown to compromise PDE5I effectiveness [14]. Our study found no significant difference in the number of diabetic patients between the treatment failure group and the control responder group. However, there were statistically significant differences observed in poorly controlled diabetes mellitus, as indicated by HbA1c levels. This implies that higher HbA1c values are indicative of inadequately managed DM and a heightened susceptibility to vascular and neuropathic complications associated with diabetes.

In our study, we meticulously defined inclusion and exclusion criteria, focusing on patients who had consistently taken PDE5Is for at least eight weeks to ensure a relevant and reliable analysis of the data, given that patient compliance is critical for the effectiveness of treatments, including PDE5Is, used in managing erectile dysfunction. Studies have shown wide variance in PDE5I discontinuation rates, from 14% to over 80%, attributed to various factors such as medical complications, inadequate results, severe side effects, and personal reasons like changes in libido, relationship issues, and dependence on medication for erectile function. Additional barriers like fear of side effects, medication accessibility, and financial burden also play a role. Moreover, some patients cease medication due to a lack of sexual spontaneity [9,15-18].

Additionally, in our study, the exclusion criteria were rigorously applied to ensure the safety and applicability of the findings. Patients contraindicated for the use of PDE5Is due to recent cardiovascular events such as stroke or myocardial infarction, those with significant hypotension or uncontrolled hypertension, unstable angina, advanced cardiac failure, severe hepatic insufficiency, or end-stage renal disease necessitating dialysis [19], were systematically excluded from the analysis. This was done to mitigate the risk of adverse events and to uphold the integrity of the study results.

Based on our analyses, we identified low FT and high SHBG as factors that increased the likelihood of PDE5I treatment failure. As testosterone balance is important for erectile and sexual function, low FT was expected to correlate with ED severity, as confirmed in prior investigations. Also, our results show no statistical significance on the TT level, but in FT and SHBG, they were significant. Relying solely on TT levels is not sufficient for patients with ED, particularly in individuals aged over 60 who frequently exhibit increased SHBG levels. Morgado et al. estimated that one-fourth of men over 60 years old might be incorrectly diagnosed with normal gonadal function due to normal TT levels, while they actually have reduced calculated free testosterone (cFT). It is imperative to update the current diagnostic procedures for male hypogonadism to avoid these inaccuracies. It is recommended that clinical guidelines be expanded to include FT evaluations or cFT estimations to ensure low FT levels are identified even in cases where TT levels are within the normal range, particularly in the elderly population with ED [20].

Although these findings are highly informative, they need to be interpreted in light of the study's limitations. Specifically, this was a retrospective with an age-matching case-control study. In addition, there is no clear definition for PDE5I treatment failure in the literature; besides, it was not possible to ascertain the exact time of treatment failure after the PDE5I initiation due to infrequent follow-up visits (typically at six-monthly intervals, as is the standard in our hospital). Also, the daily dose of Tadalafil (5 mg) was not evaluated in that group of patients. Moreover, other risk factors (such as psychological factors, partner-related factors, and relationship quality) were not considered. Further research is required to obtain a more comprehensive picture of ED and the reasons behind treatment failure. On the other hand, as all our patients receive PDE5I for free from our hospital pharmacy, treatment cost and affordability were not included in the analysis.

## Conclusions

Men diagnosed with ED are more likely not to benefit from PDE5I treatment if they have high HbA1c levels and/or low free testosterone and/or high SHBG. Relying solely on TT levels is not sufficient for patients with ED, particularly in individuals aged over 60 who frequently exhibit increased SHBG levels.

Therefore, management of these conditions should be a priority in this cohort, which may improve treatment effectiveness. Further prospective randomized studies are nonetheless required to assess the effects of other factors that were not incorporated into our analyses.
